# Beyond virology: environmental constraints of the first wave of COVID-19 cases in Italy

**DOI:** 10.1007/s11356-021-12878-x

**Published:** 2021-02-23

**Authors:** Christian Mulder, Erminia Conti, Salvatore Saccone, Concetta Federico

**Affiliations:** Department of Biological, Geological and Environmental Sciences, University of Catania, Via Androne 81, 95124 Catania, Italy

**Keywords:** COVID, Fine particulate matter, PM_2.5_, Viral infection, SARS-CoV-2

## Abstract

Global warming and air pollution affect the transmission pathway and the survival of viruses, altering the human immune system as well. The first wave of the COVID-19 pandemic dramatically highlights the key roles of climate and air chemistry in viral epidemics. The elongated form of the Italian peninsula and the two major islands (the largest in Europe) is a perfect case study to assess some of these key roles, as the fate of the virus is mirroring the industrialization in the continental part of our country. Fine particulate matter (PM_2.5_), geography, and climate explain what is happening in Italy and support cleaner air actions to address efficiently other outbreaks. Besides the environmental factors, future works should also address the genetic difference among individuals to explain the spatial variability of the human response to viral infections.

## Introduction

Biodiversity provides benefits to human health, and the abrupt loss of so many species and ecosystems threats the thin balance between ecosystem functioning and human wellbeing (MEA 2005; Mulder et al. 2015). On the one hand, belowground biodiversity is recognized as a key environmental buffer because among others it suppresses disease-causing soil pathogens (Wall et al. 2015). On the other hand, aboveground biodiversity acts at several levels of the organization by providing alternative paths and regulation services for airborne pathogens (Morens and Fauci 2020).

Biodiversity loss, disruption of entire ecosystems, and climate changes pose a tremendous policy challenge: while keeping UN actions such as the Sustainable Development Goals and the Paris Climate Agreement running, with pandemic containment in sight, the world will face a drumbeat of climate-driven adaptation crises (IPBES 2019; Phillips et al. 2020). And besides climate, conservation biology, and social sustainability, it is environmental chemistry that matters the most.

Dependent on the biochemical characteristics of pathogens, the environment, and the specific urban population being studied, humans may or may not be exposed to a specific contagion, this contagion may or may not lead to a viral disease, and this viral disease may or may not have (lethal) effects. Toxicokinetic effects will occur as a consequence of a number of external (outside organisms) and internal (within organisms) transport processes, but to our knowledge, the role of environmental chemistry is highly underestimated and the results biased towards toxicology. Although the current pandemic demands high-resolution mechanistic models to forecast and contain the contagion, it is not yet our aim to investigate the possible biochemical implications but we wish to unravel possible macroecological patterns.

Recent macroecological predictions show a remarkably high amount of new viral sharing events in South-East Asia (Carlson et al. 2020a). As a matter of fact, on December 31, 2019, a novel coronavirus disease, the COVID-19, was identified by the Wuhan Municipal Health Commission, Hubei Province, China, where a cluster of cases of pneumonia was reported (Huang et al. 2020). On March 11, 2020, the World Health Organization declared the coronavirus as pandemic (WHO 2020). Most Coronaviridae are known to cause severe respiratory syndromes, and the extent to which they are climate-driven is debated. For instance, pandemic influenza displayed little of the climate patterns that seasonal influenza does (Carlson et al. 2020b). However, the majority of eco-epidemiological studies do not account for environmental quality.

The Chinese province of Hubei, where everything was thought to begin, is located in the hotspot of new viral sharing events of the study by Carlson et al. (2020a), who used a computational model accounting for climate and land use. But the aforementioned predictions accounting for global warming and land use change do not predict a high occurrence of viral sharing events in Italy, although the area of Hubei is climatologically comparable to the middle of Northern Italy (in January 2020, the average temperature was 5 °C for both the cities of Wuhan and Milan and the overall absolute humidity was 6 and 5%, respectively).

Italy was the first EU country facing the epidemic and was able immediately to apply strong containment measure in all the regions of the country. Meanwhile, with 2,028,354 patients, the total number of COVID-19 cases in Italy is more than two million of inhabitants (http://opendatadpc.maps.arcgis.com/apps/opsdashboard/index.html#/b0c68bce2cce478eaac82fe38d4138b1 last accessed December 25, 2020). Phylogenetic analysis on the complete genomic sequences from the three first patients discovered in Italy suggested multiple SARS-CoV-2 introductions in the fourth trimester of 2019 in Italy, and in Europe, or virus evolution during circulation (Giovanetti et al. 2020a, b; Stefanelli et al. 2020). Further data demonstrated a simultaneous circulation of SARS-CoV-2 in the North and in the South of Italy (La Rosa et al. 2021), despite the containment measures applied in all the country, and novel evidence supports a very rapid evolution of the virus, with variants among others in South Africa, Spain, and the UK (Hodcroft et al. 2020; Le Page 2020; Rambaut et al. 2020).

**Fig. 1:**
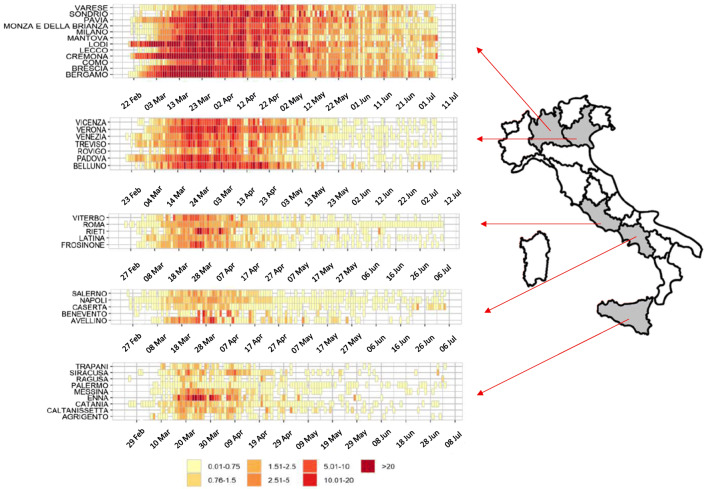
Examples of time series of COVID cases in five regions during the first wave in Italy. Source: https://www.epicentro.iss.it/coronavirus/ (incidence of COVID cases per 100,000 inhabitants, figures modified and adapted)

A very different spread rate was observed in the different regions of Italy during the “first wave” (Fig. 1). To better understand the reason why the two geographic macroregions exhibited significantly different prevalence of COVID-19, we wish to investigate what happened during the first semester of 2020 in Italy, focusing on environmental variables and infected inhabitants. Commonly used proxies for air quality relevant to human health and epidemiology (Dominici et al. 2006; WHO Europe 2013; Wu et al. 2020) refer to particulate matter (PM), i.e., to the mass concentration of particles with a diameter of less than 2.5 μm (hereafter: PM_2.5_). Viruses are not commonly airborne and are likely attached to suspended particles such as fine particulate (e.g., Yang et al. 2011; Després et al. 2012), and the inactivation of viruses is known to be under the influence of changes in temperature and relative humidity (Després et al. 2012). Hence, we wish to investigate the extent to which climate, urban topology, and fine particulate are major environmental predictors for the COVID-19 diffusion during the first wave in Italy.

## Material and methods

As the initial phase of this research, we propose a project with the *overall objective* of addressing the aforementioned definition and associated question, by compiling the existing knowledge base and available (bio)monitoring data in Italy on particulate exposures and their geographical and climatological patterns, the latter for selected COVID-19 case study areas. To achieve this, data web searches were conducted with machine learning using derivations of the following Italian keywords: *ministero*, *latitudine*, *longitudine*, *altitudine*, *radiazione solare*, ENEA, *particolato fine*, PM_2.5_, *precipitazione*, *temperatura*, *grado*, *urbanizzazione*, 2019, 2020, and the investigated urban domains (Alessandria, …, Vicenza). Then, building on that base, we will develop an integrated framework for quantifying the co-relationships between particulate and contagion and related effects on the human populations. This framework will likely be tiered and will comprise a set of useful and practical tools, to be selected based on-site locations, the geographical data, and the particulate.

Based on 69 large cities and 13 large towns in Italy, the average solar radiation (January–June) was computed according to the ENEA-SOLTERM model, the average altitudes of these urban domains and their population density in clusters as defined by ISTAT in 2019 were gathered by data mining, the measured PM_2.5_ air concentration, the latitude and longitude the temperature, and the rainfall in the same domains (predictive variables) were downloaded from the GHS Urban Centre Database (Florczyk et al. 2019), while all the 204,234 COVID-19 cases (response variable) were downloaded from the Italian ‘Dipartimento della Protezione Civile’ dataset (http://opendatadpc.maps.arcgis.com/apps/opsdashboard/index.html#/b0c68bce2cce478eaac82fe38d4138b1 last accessed December 23, 2020). A curve estimation model was fitted to estimate the parameters of regression and the coefficient of significance using SPSS software (vers. 21). A heat map analysis was performed using XLSTAT 2019.1.1 with the aim to cluster the contributions of the environmental variables and the COVID-19 cases in descending order of importance to the total variation of the dataset. We visualized further the PM_2.5_ frequency distribution using violin plots as realized with the “ggplot2” program by the “geom_violin ()” utility in R-3.5.1. We used the average of all 82 urban domains as a cut-off value, and different PM_2.5_ plots were split according to the means of solar radiation, annual rainfall, and air temperature (4.25 kWh/m^2^, 840 mm/year, and 14.7 °C, respectively).

## Results and discussion

Data from Italy were statistically evaluated in order to identify environmental, climatic, or other non-geographic parameters as possible causes of the diversified spread of the SARS-CoV-2 virus in the different urban domains. First of all, we divided the entire sample of 82 areas into four groups (A, B, C, and D) according to the detected COVID cases. The first group (A) included 22 cities with the largest number of COVID cases (more than 3000 cases in the first semester of 2020, with an average of deceases with respect to the total population of 0.732%); the second (B), the third (C), and the fourth (D) included each of them 20 cities with a decreasing number of cases (between 1000 and 3000 cases with 0.149% deceases the second, between 450 and 1000 cases with 0.065% deceases the third, and less than 450 cases with 0.016% deceases the fourth population group). Each group was compared to the other three groups in order to evaluate statistical differences in the distribution of the investigated parameters (Table 1). Some parameters are clearly not correlated with or related to COVID, like the population density (hereafter: urbanisation) and the average altitude. This is not unexpected, given that for the first parameter, the population densities and the related degree of urbanisation in many southern cities can be remarkably high (for instance, taking into account only the first two population groups, Milan (A) with Naples (B) and Bergamo (A) with Bari (B) have statistically undistinguishable numbers of inhabitants per square km) and for the latter parameter, the distribution across hills of some Italian cities is highly scattered (for instance, Triest ranges from 0 up to 674 m a.s.l. and Brescia ranges from 149 up to 874 m a.s.l.).

**Table 1:**
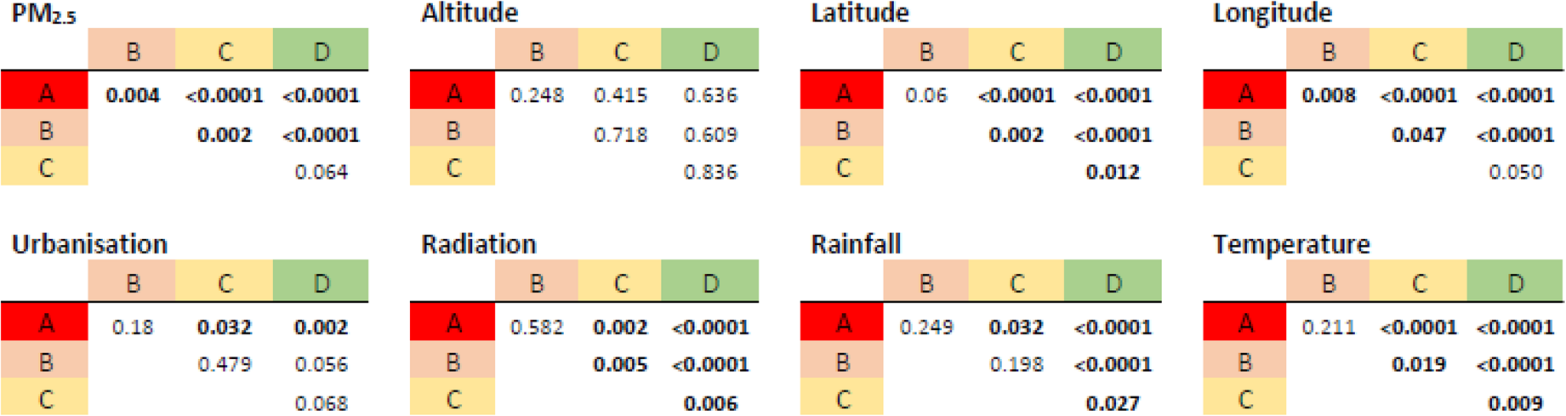
Statistical significances of the investigated parameters between different populations groups (A, B, C, and D). The population, consisting of data from 82 urban domains across Italy, was divided in groups with the same size (22 cities in the 1^st^ group, here as A, and 20 cities in the 2^nd^, the 3^rd^, and the 4^th^ group, here as B, C, and D, respectively) according to the number of persons infected during the first semester of the COVID pandemic

**Fig. 2:**
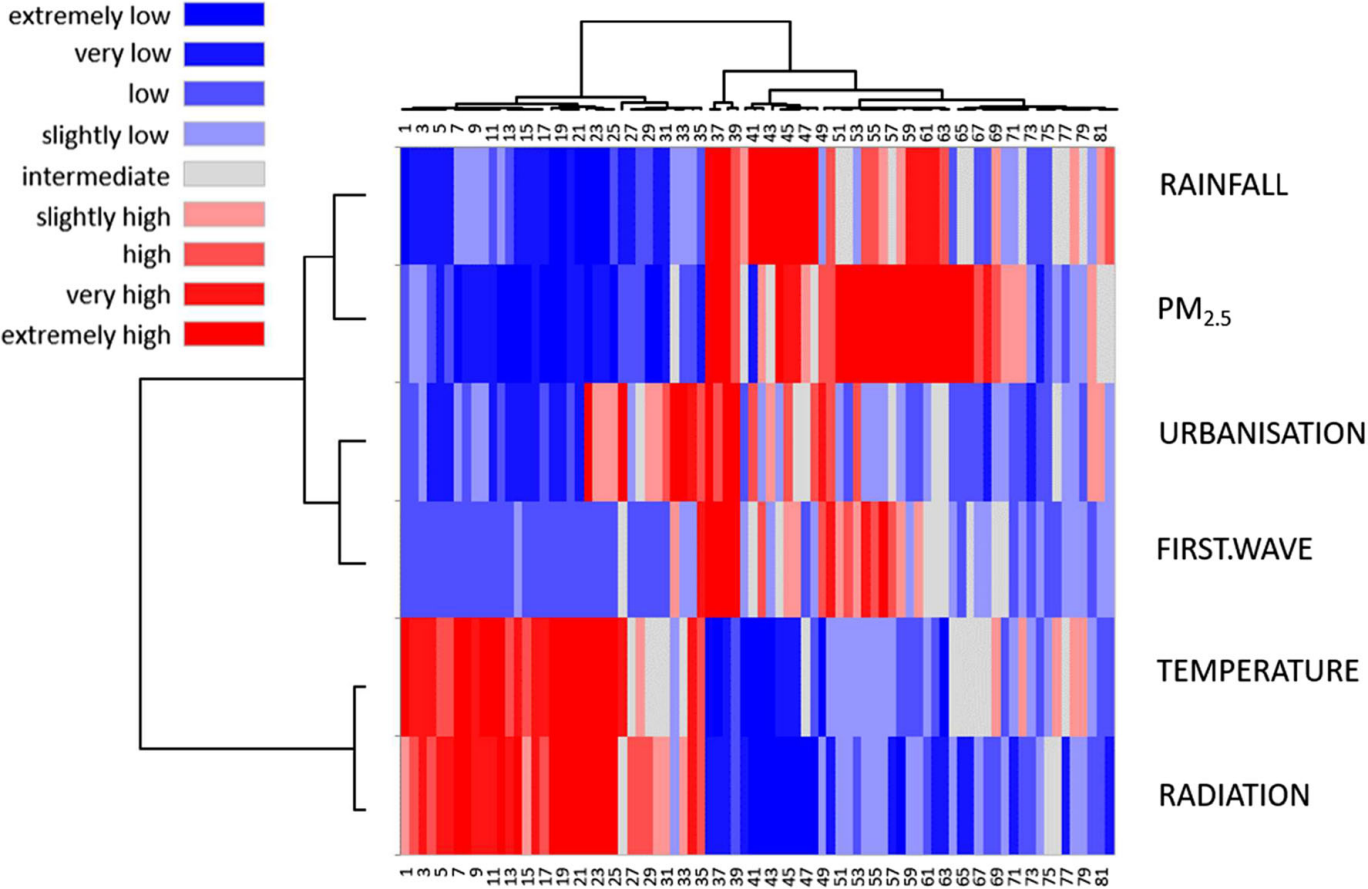
Heat map of the COVID cases and the environmental variables for 82 urban domains (numbered as in Table 2). More details in the text

All the parameters that showed a very high level of differences were statistically evaluated to see if the contagion is mirroring some of the environmental parameters. To unravel the actual relationships between environment and contagion, i.e., the first wave cases, we performed a direct comparison between urban domains and non-geographical parameters using a heat map. Only in this way, we will be able to check if patterns between the separate urban domains, stored in 82 rows in our dataset, and their environmental parameters, stored in 6 columns, become recognizable despite their location. Hence, our heat map will show simultaneously if clustering occurs, as urban domains and parameters are clustered independently: this brings similar cities close to each other in rows and similar parameters close to each other in columns. To increase the robustness and the readability of our statistical analysis, latitude, longitude, and altitude were not taken into consideration, as we focused for each urban domain on its five environmental predictors (rainfall, particulate (PM_2.5_), population density (urbanisation), temperature, and solar radiation) and the response (first wave COVID cases). In Fig. 2, we can clearly visualize different patterns, where blue stands for low values, grey stands for intermediate values, and red stands for high values, making rectangular patterns evident. If we focus on the upper dendrogram, we see a remarkable dichotomy, and if we focus on the vertical dendrogram, we see two different horizontal groups, above the scores of the rainfall, the PM_2.5_, the urbanisation and the COVID cases grouped together, and below the scores of the temperature and the radiation grouped together. If we take a closer look to the first vertical cluster, we see—besides for Florence—a striking dominance of southern cities with less than 1000 cases, in sharp contrast to the second vertical cluster, where we see a massive dominance of northern cities with often much more than 1000 cases (Table 2). More in detail, the centroid of the first vertical cluster as shown in Fig. 2 is located at 40°15′28″N and 14°42′14″E, while the centroid of the second vertical cluster is located within the Po Valley (44°47′56″N and 10°44’20″E), 606 km far from the first centroid. Excluding the two outliers (the aforementioned Florence and Terni), the two clusters overlap only between 42°46′04″N and 43°06′10″N.

**Table 2:**
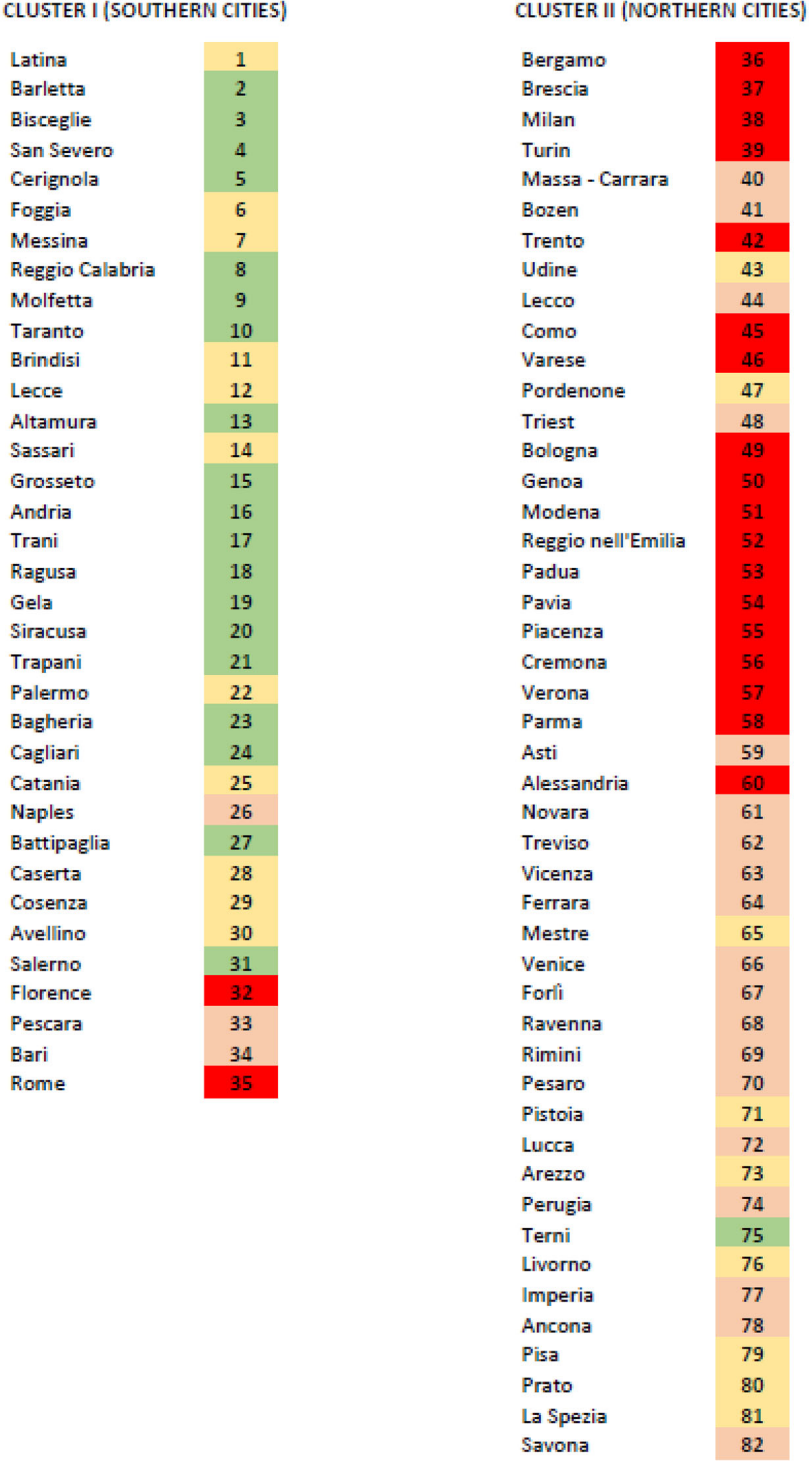
The two blocks resulting from the heat map analysis. On the left, the southern cities and on the right the northern cities, both sequenced as in Fig. 2. Colors of the site numbers according to the four previous population groups (A–D), based on the number of persons infected during the first COVID wave

The resulting patterns are clearly related to the geographical position of the 82 urban domains, even if many environmental parameters are closely correlated with each other (Table 3). Overall, at *α* = 0.05, the Pearson’s correlation coefficient between the first wave of COVID cases and the considered environmental predictors is the strongest for PM_2.5_ and cases (depicting with *r* = 0.394 a strong direct correlation), followed by longitude (a strong inverse correlation with *r* = −0.378), latitude (a robust direct correlation with *r* = 0.332), and temperature (a robust inverse correlation with *r* = −0.285). In other words, during the first wave, the cleaner the air, the less the COVID cases, etc.

**Table 3:** Correlation matrix between the investigated parameters. In bold the significant values (significance level *α* = 0.05), with the *P* values in smaller font and lower case and above the Pearson’s correlation coefficients in a larger font

Variables	LATITUDE	LONGITUDE	RAINFALL	RADIATION	TEMPERATURE	ALTITUDE	PM_2.5_	URBANISATION	FIRST_WAVE
LATITUDE	**1**	**-0.646**	**0.708**	**-0.949**	**-0.797**	0.028	**0.802**	0.127	**0.450**
		**<0.0001**	**<0.0001**	**<0.0001**	**<0.0001**	0.806	**<0.0001**	0.257	**<0.0001**
LONGITUDE	**-0.646**	**1**	**-0.437**	**0.592**	**0.538**	-0.087	**-0.635**	-0.180	**-0.467**
	**<0.0001**		**<0.0001**	**<0.0001**	**<0.0001**	0.439	**<0.0001**	0.105	**<0.0001**
RAINFALL	**0.708**	**-0.437**	**1**	**-0740**	**-0.736**	0.128	**0.521**	**0.233**	**0.437**
	**<0.0001**	**<0.0001**		**<0.0001**	**<0.0001**	0.253	**<0.0001**	**0.035**	**<0.0001**
RADIATION	**-0.949**	**0.59**2	**-0.740**	**1**	**0.816**	-0.044	**-0.712**	-0.152	**-0.371**
	**<0.0001**	**<0.0001**	**<0.0001**		**<0.0001**	0.696	**<0.0001**	0.172	**0.001**
TEMPERATURE	**-0.797**	**0.538**	**-0.736**	**0.816**	**1**	**-0.246**	**-0.564**	-0.164	**-0.452**
	**<0.0001**	**<0.0001**	**<0.0001**	**<0.0001**		**0.026**	**<0.0001**	0.140	**<0.0001**
ALTITUDE	0.028	-0.087	0.128	-0.044	**-0.246**	**1**	-0.151	-0.022	0.148
	0.806	0.439	0.253	0.696	**0.026**		0.176	0.847	0.185
PM_2.5_	**0.802**	**-0.635**	**0.521**	**-0.712**	**-0.564**	-0.151	**1**	0.132	**0.527**
	**<0.0001**	**<0.0001**	**<0.0001**	**<0.0001**	**<0.0001**	0.176		0.237	**<0.0001**
URBANISATION	0.127	-0.180	**0.233**	-0.152	-0.164	-0.022	0.132	**1**	**0.609**
	0.257	0.105	**0.035**	0.172	0.140	0.847	0.237		**<0.0001**
FIRST_WAVE	**0.450**	**-0.467**	**0.437**	**-0.371**	**-0.452**	0.148	**0.527**	**0.609**	**1**
	**<0.0001**	**<0.0001**	**<0.0001**	**<0.0001**	**<0.0001**	0.185	**<0.0001**	**<0.0001**	

**Fig. 3:**
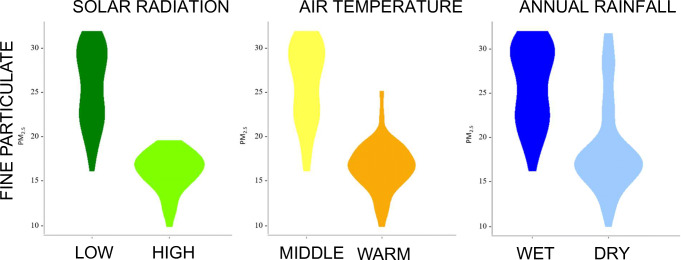
Frequency distributions of the total concentration of fine particulate matter (PM_2.5_, expressed in μg/m^3^) calculated over our 82 urban domains according to climatological predictors

The frequency distributions of PM_2.5_ (Fig. 3) according to radiation (low vs. high), temperature (middle vs. warm), and rainfall (wet vs. dry) show a remarkable dichotomy between the more continental, northern, temperate, and humid part of Italy and the Mediterranean climate of the South and the major islands (Sardinia and Sicily). In the South, the PM_2.5_ concentration averages 15.99 ± 2.33 SD, much less than in the North (31.86 ± 4.37 SD), mirroring the number of COVID-19 cases recorded in Italy between January 1 and June 30, 2020, that average 634 ± 1036 SD in the South and 3873 ± 4622 SD in the North (*P* < 0.01).

Regarding the climatological predictors, the Coefficient of Variation (CV = 100SD/mean) is very different for the North and the South (the two major Italian islands included), being it equal to CV_Low_ = 1.89% vs. CV_High_ = 4.14% (radiation), CV_North_ = 15.52% vs. CV_South_ = 7.41% (temperature), and CV_North_ = 28.63% vs. CV_South_ = 14.77% (rainfall). Regarding the mortality rates, in both the North and the South, the number of deaths is (obviously) a direct function of the number of infected patients, but the allometrical distributions of dead vs. infected inhabitants are remarkably different. In the South, the linear regression slope for mortality rates is 0.099, while in the North it is much steeper, being the slope equal to 0.169; in other words, in the North, the possibility to pass away after getting contaminated during the first COVID-19 wave was about 70% higher than in the South.

In our 82 Italian urban domains, the COVID-19 cases in the first six months of 2020 and the latitude and the PM_2.5_ are the variables most closely correlated with each other. Wu et al. (2020) already discovered that an increase in PM_2.5_ of 1 μg/m^3^ is directly associated with an increase in the COVID-19 death rate of 15% in the USA. One of the hypotheses that could be investigated should be the possibility of the particulate matter to carry the virus into the environment and keep it viable for a longer time. This makes our results highly relevant for extensive air quality monitoring to improve the urban sustainability and hence the human well-being.

Our results indicate the air quality (PM_2.5_) as one of the more relevant parameters influencing the spread of the virus during the first wave, justifying the high level of variability on the viral dissemination observed in the different geographical areas across Italy. Besides the different rate of infection among regions, inside each area, other factors influencing the human response to the viral infection and the severity of disease should be considered. Some were ascribed to subjects with the COVID-19 disease beside cohabiting family members not showing neither disease symptoms nor virus detection (unpublished data). This suggests the onset of COVID-19 disease clearly related to a genetic difference among individuals, with polymorphisms of human genes related to the life cycle of the SARS-CoV-2 virus in the human cells, such as *ACE2* and *TMPRSS2* (Murray et al. 2020; COVID-19 Host Genetics Initiative 2020), playing a pivotal role.

Finally, for future research, we are going to correlate COVID-19 cases and case-fatality rates in different countries and regions with specific DNA polymorphisms, to understand better the SARS-CoV-2 biology and epidemiology joining a genetic and ecological point of view (Morens and Fauci 2020). Here, we demonstrated within an ensemble of 82 urban domains that by entering geo-tagged climatological and chemical parameters into the eco-epidemiological database, it will become possible to unravel challenging interactions between pandemics, urban pollution, and global warming, linking the epidemiological knowledge gleaned from medical records with a biophysical interpretation at urban and regional scales for the second and other waves.

## Data Availability

The authors used only publicly available official datasets. Response data can be found at http://opendatadpc.maps.arcgis.com/apps/opsdashboard/index.html#/b0c68bce2cce478eaac82fe38d4138b1 (COVID), and predictive data can be found at http://cidportal.jrc.ec.europa.eu/ftp/jrc-opendata/GHSL/GHS_STAT_UCDB2015MT_GLOBE_R2019A/V1-2/ (urban air quality) and http://www.solaritaly.enea.it/CalcRggmmOrizz/Calcola1.php (mean solar radiation in kWh/m^2^).
